# Compatible Pollinations in *Solanum chacoense* Decrease Both S-RNase and S-RNase mRNA

**DOI:** 10.1371/journal.pone.0005774

**Published:** 2009-06-03

**Authors:** Bolin Liu, David Morse, Mario Cappadocia

**Affiliations:** Institut de Recherche en Biologie Végétale (IRBV), Biology Department, University of Montreal, Montreal, Québec, Canada; University of Melbourne, Australia

## Abstract

Gametophytic self-incompatibility (GSI) allows plants to block fertilization by haploid pollen whose S-allele constitution matches one of the two S-alleles in the diploid styles. GSI in *Solanum chacoense* requires a stylar S-RNase, first secreted from cells of the transmitting tract then imported into incompatible (self) pollen tubes. However, the molecular mechanisms allowing compatible pollen to evade S-RNase attack are less clear, as compatible pollen tubes also import S-RNase. Using styles of the same age and size in order to lower the degree of inter-style variability in S-RNase levels, we observe reduction of up to 30% of the total non-self stylar S-RNase *in vivo* during compatible crosses, whereas no degradation of self S-RNases is detected. This marked difference in stylar S-RNase levels dovetails with measurements of pollen-specific Lat52 mRNA, which decreases four-fold in incompatible compared to compatible crosses. Unexpectedly, we also find evidence for a reciprocal signaling mechanism from compatible pollen to the cells of the transmitting tract that results in a roughly three-fold decrease in S-RNase transcript levels. These findings reveal a previously unsuspected feedback loop that may help reinforce the compatible reaction.

## Introduction

Self-incompatibility (SI) is a genetically inherited prezygotic barrier widespread among flowering plant species that promotes outbreeding by allowing the pistil of a hermaphrodite flower to discriminate between genetically related (self) and unrelated (non-self) pollen [1], [2]. This cell-cell recognition mechanism involves finely tuned interactions of gene products secreted by specialized cells of the pistil with complementary proteins expressed inside the pollen tube. As a result of these interactions, self-incompatible plants recognize and block growth of self or genetically identical (incompatible) pollen tubes, and thus only non-self (compatible) pollen is allowed to accomplish fertilization. When the compatibility of the pollen is determined by its haploid genotype, SI is termed gametophytic (GSI). GSI is the most widespread of the SI systems [3], and is mainly controlled by elements of a multigenic S-locus, inherited as a single segregating unit that includes, among other genes, the highly polymorphic male and female determinants to SI. Variants of the entire S-locus have been termed S-haplotypes, whereas variants of the individual polymorphic genes within the S-locus are called alleles [4].

In the Solanaceae, Rosaceae and Plantaginaceae, the pistillar determinant to SI is a glycoprotein with RNase activity (S-RNase) expressed in the extracellular matrix of the pistil [5]–[7]. S-RNases show a pattern of highly conserved (C) and highly variable (HV) regions in the primary sequence, whose functions are now reasonably clear. Thus, the hypervariable regions (two in the Solanaceae and one in the Rosaceae) are involved in allelic recognition [8], [9] and as such are under positive selection pressure [10]. The constant regions C2 and C3 each contain an histidine residue essential for RNase activity [11], while C1, C4 and C5 are now thought to be involved in stabilizing the three dimensional structure of the S-RNase [12], [13].

S-RNases presumably exert their cytotoxic action inside the incompatible pollen tubes by degrading pollen tube RNA in an S-haplotype-specific manner, as S-RNases mutated at the essential histidine residues or lacking one of these histidines lose their RNase activity and are unable to reject incompatible pollen [14], [15]. Furthermore, S-RNases have been shown to enter pollen tubes in S-haplotype-independent manner, as assessed by immunolocalization studies [16], [17]. This implies that the SI mechanism must involve the ability of pollen tubes to inactivate any non-self S-RNase, whereas self S-RNases are somehow protected from inactivation. In turn, the inhibition of RNase activity for all but the self S-RNases implies recognition of a region common to all S-RNases [18]. To date, however, none of the conserved regions appears able to play this common recognition role, with even the most attractive possibility (the conserved C4 region with its three charged amino acids) apparently uninvolved [13].

The S-locus gene product expressed in pollen has proved much more difficult to isolate than the pistillate component, but has finally been identified as an F-box protein, termed *S*-locus F-box gene (SLF) in the Solanaceae (*Petunia*), or *S*-haplotype-specific F-box gene (SFB) in the Rosaceae (*Prunus*) (for reviews see [18], [19]. F-box proteins usually constitute a subunit of the SCF complex also containing Skp1, Cullin-1, and Rbx1, where their role is to confer target specificity to proteins to be degraded by the 26S proteasome. Such process involves activation, conjugation and ligation of polyubiquitin chains to specific substrates, and is catalyzed by the E1, E2 and E3 enzyme complexes, respectively [20], [21]. Surprisingly, several genes related to SLF/SFB and called *SLF-like* genes have also been found at the S-locus of the three families mentioned above (see [22], [23]). The function of these genes is presently unknown, although it has already been demonstrated by transgenesis that they cannot substitute for the authentic SLFs with regard to the S-specificity of pollen [22]. Similarly to what was observed with the S-RNases, SLF/SFB proteins also display a pattern of conserved and highly variable regions in the primary sequence, whose functions are now being investigated. Thus, apart from a common N-terminal F-box motif, the primary sequence of these proteins has four conserved regions [23], and two variable regions in both *Prunus* [24]–[27] and *Petunia inflata* [22], [28]. In the latter species, sequence comparison among three SLF alleles and six SLF-*like* genes has uncovered three regions specific to SLFs, and the two variable regions are located inside two of these SLF-specific regions. Interestingly, the SLF-specific regions are embedded in three functional domains whose secondary structure predicts loops potentially involved in protein-protein interactions [22].

Other products of genes unlinked to the S-locus have also been shown to be involved in the SI phenomenon [19], [29], [30]. These proteins include HT-B, a small non-polymorphic asparagine-rich protein [31], [32] produced by the pistil that enters pollen tubes in S-haplotype independent manner [19]. Interestingly, antisense or RNAi experiments have shown that suppression of HT-B results in suppression of pollen tube inhibition in incompatible crosses, although S-RNases levels are not affected [31], [32]. In addition to HT-B, other factors able to bind to S-RNases *in vitro* have also been thought to be involved in pollen rejection [29], [33]. Of these factors, however, only a 120K protein has been shown to be directly involved in S-RNase-based SI, as its suppression by RNAi results in S-haplotype independent suppression of pollen tube inhibition in incompatible crosses.

The identification of the pollen S-gene product as an F-box protein has reoriented research attention from the incompatible to the compatible reaction, as it is during compatible pollinations that S-RNase degradation would be most coherent with the known functions of F-box proteins. Two models, described as degradation and sequestration models, have recently been proposed to explain how non-self pollen tubes escape the cytotoxic activity of the S-RNase. In the degradation model, S-RNases enter the pollen tube cytoplasm but interact differently with SLFs during compatible and incompatible pollinations. In particular, all non-self S-RNases are recognized, targeted for ubiquitination and subsequently degraded by the 26S proteasome [22], [30], [34]–[37]. In an effort to identify the lysine residues constituting the possible targets for ubiquitination in the Solanaceae, a number of transgenic S-RNases have been tested. In one, the conserved lysine in the C4 region of the *Solanum chacoense* S_11_-RNase [13] was substituted with arginine. Since this substitution did not disrupt the SI behavior of the transgenics, it was concluded that this conserved lysine is not the primary target for ubiquitination. More recently, similar studies with *P. inflata* have implicated six lysines near the C-terminal in S-RNase degradation [34]. Interestingly, further support for the degradation model has been provided by the recent discovery of a novel type of an E3 ubiquitin ligase complex [30], where a RING finger protein called SBP (for S-RNase-binding protein) first described in *P. hybrida* [38], substitutes for the Skp1 and Rbx1 subunits in the classic SCF complex and binds to SLF. *In vitro*, SLFs bind more strongly with non-self S-RNases than with their cognate S-RNase [30]. This important result provides a biochemical explanation for the intriguing phenomenon called competitive interaction, in which pollen expressing two different SLFs (S-heteroallelic pollen) are accepted by any pistil [22], [39].

Unlike the degradation model, the sequestration model proposes that instead of direct import into the pollen tube cytoplasm, both self and non-self S-RNases are directed into a vacuolar compartment sequestered from the cytoplasm. The vacuolar compartment is somehow disrupted if the pollination is incompatible, releasing S-RNases into the cytoplasm of the self pollen tube, where they exert their cytotoxic activity leading to the inhibition of pollen tube growth [16]. According to the sequestration model, it is HT-B and not the S-RNase that is degraded (by an as yet unidentified protein) in compatible pollen tubes, whereas in incompatible crosses HT-B remains functional, causing the disruption of the vacuolar compartment and the subsequent release of the sequestered S-RNases into the cytoplasm [16], [19], [29].

We have recently developed a method for the quantification of S-RNase levels in single styles, which has allowed the S-RNase threshold required for rejection of *S_11_* and *S_12_* pollen by the S_11_- and S_12_-RNases, respectively, to be determined [40], [41]. Style-by-style measurements yield not only average S-RNase levels but also estimates of the variation between styles and are thus superior to those using groups of styles that only determine averages. Indeed, while S-RNases are quite abundant in the style [42], the amount of S-RNase accumulated in different styles of the same plant can vary by over 20-fold [41]. The large variation in S-RNase levels between styles has been the most important factor hampering direct assessment of differences in S-RNase levels after compatible and incompatible pollinations [34]. In this study, we performed style-by-style analyses to determine the levels of S-RNase after compatible and incompatible pollinations. In particular, we found that selecting styles of identical size and stage of development was crucial in reducing variation in S-RNase levels between styles. Our results provide additional support for the degradation model by providing direct evidence that S-RNases are indeed degraded *in vivo*. Furthermore, our studies show a substantial reduction in the amounts of stylar S-RNase transcripts after compatible but not after incompatible pollinations, suggestive of a previously unsuspected feedback mechanism acting to reinforce the compatibility process.

## Results

### Stylar age and size contribute to inter-style variation in S-RNase levels

**Figure 1:**
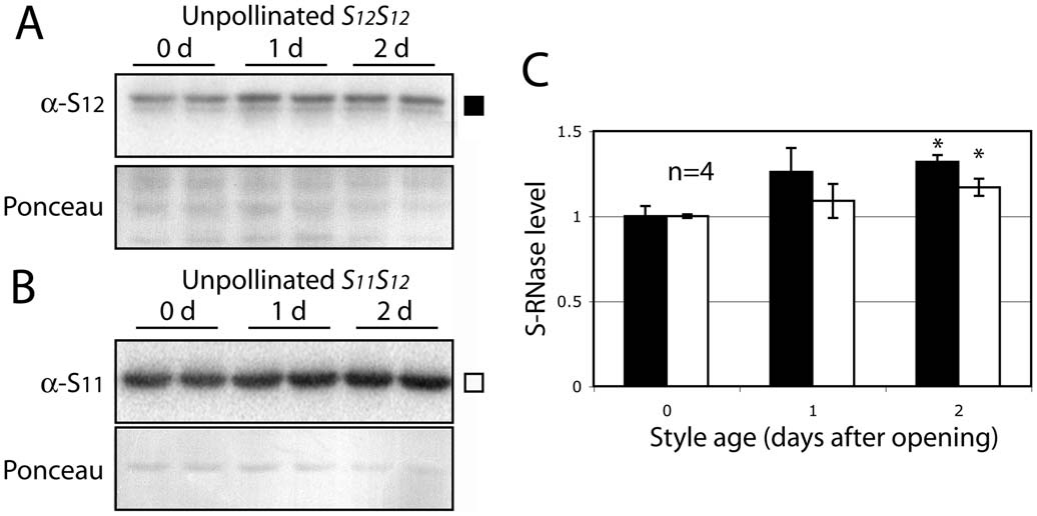
Individual unpollinated styles have similar levels of S-RNase when selected by size and age. The levels of S_12_-RNase in the homozygous *S_12_S_12_* genetic line 2548 (A) and S_11_-RNase in the *S_11_S_12_* line 314 (B) were measured by Western blot analysis in styles selected to be of the same size and age. Each lane contains the entire protein extract from one individual style. Antibody reaction was monitored using a radio-iodinated secondary antibody, quantified using a PhosphorImager and reported relative to day 0 (C). Styles at day 2 show significantly higher levels of both S_11_- and S_12_-RNase than styles at day 0. Values are means±s.d. of S_11_ and S_12_-RNase levels from 2 independent experiments. Asterisks are significantly different (p<0.05, T test, n = 4).

Previous attempts to document a decrease in S-RNase during compatible crosses in *S. chacoense* have been hampered by a roughly 70% difference in S-RNase levels between styles [41]. This variability clearly precludes measurement of the small changes in stylar S-RNase levels expected to occur following pollination, so techniques to reduce this variation were tested. The possibility that differences in the size of the styles might contribute to differences in S-RNase levels was tested by selecting individual unpollinated styles of identical size (same length and diameter) for measurement. While this selection process placed a severe restriction on the number of styles that could be tested at any one time, the average inter-style variability in measured S-RNase levels was indeed drastically reduced, typically below 10% (Figure 1). The size of the style thus appears to be a major contributor to the variability observed previously. A second factor, the age of the flower, also contributes to changing levels of S-RNase [8], with increasing enzyme levels observed in flowers from anthesis to at least two days after flower opening (Figure 1). To standardize these two factors, all open flowers were first removed from a plant the afternoon preceding the experiments. Flowers open during the early morning, so the next day, freshly opened flowers (corresponding to time zero) were either pollinated or left as controls. All S-RNase measurements were made the following day, at twenty-four hour post-pollination, on styles carefully selected to be of identical length and diameter.

### Compatible crosses result in decreased stylar S-RNase levels

**Figure 2:**
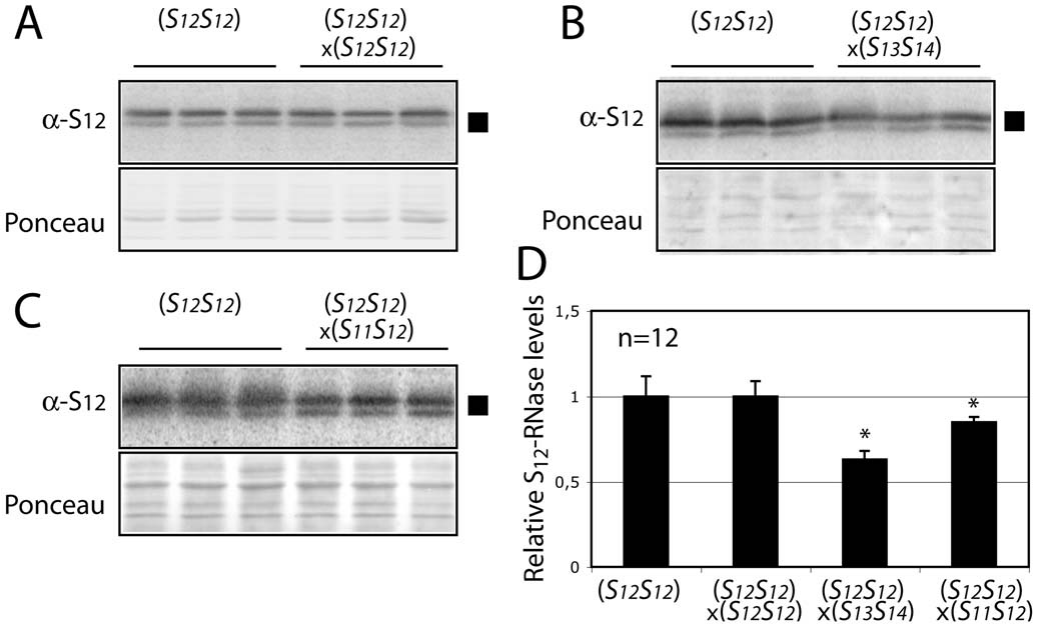
Stylar S-RNase levels decrease in compatible but not incompatible crosses. The levels of S_12_-RNase in size-selected and age-matched styles from the homozygous *S_12_S_12_* line 2548 plants were measured after pollinations with fully incompatible (self) pollen (A), fully compatible pollen from the *S_13_S_14_* line 582 (B) and semi-compatible pollen from the *S_11_S_12_* line 314 (C). Each sample contains the entire protein extract from one individual style. Levels of S-RNase were quantified by PhosphorImager and reported relative to unpollinated *S_12_S_12_* styles (left lanes in each panel) (D). Values are means±s.d. of S_12_-RNase levels from 2 independent experiments. Asterisks are significantly different (p<0.05, T test, n = 12).

To determine if the S-RNase levels could be affected by the type of pollination, styles from the homozygous *S_12_S_12_* plant line 2548 were pollinated with pollen from either a fully incompatible (homozygous *S_12_*), fully compatible (*S_13_S_14_*), or semi-compatible (*S_11_S_12_*) individuals. We found that the S_12_-RNase levels measured in unpollinated styles remain unchanged after incompatible crosses, but show a decrease of 37% after fully compatible pollinations (Figure 2). These data are consistent with the idea that pollen compatibility is associated with S-RNase degradation. Interestingly, the reduction in S_12_-RNase in styles after semi-compatible pollinations is less marked, as might be expected given that only half the pollen load has a compatible (*S_11_*) haplotype.

**Figure 3:**
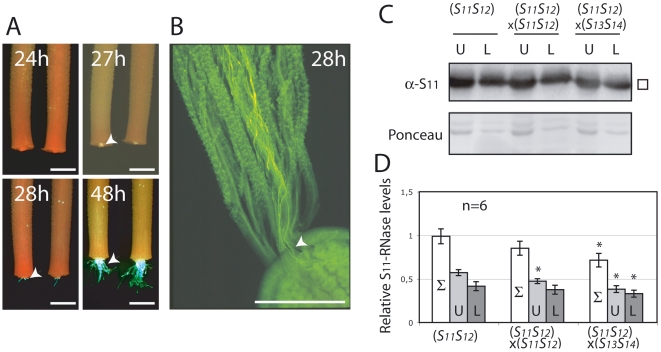
S-RNases are not translocated within the style during compatible crosses. The position of pollen tubes from transgenic *S_11_S_13_* plants expressing the fluorescence marker GFP (white arrowheads) within the styles of *S_12_S_12_* line 2548 plants was visualized by fluorescence microscopy at various times post pollination. Styles were harvested five hours post-pollination, cultured *in vitro* and observed by fluorescence microscopy (A). Pollen tubes grown *in situ* and visualized by aniline blue can also be seen to enter the ovarian region (white arrowheads) 28 hours after pollination and observed by fluorescence microscopy (B). All scale bars are 1 mm. Levels of S_11_-RNase in the upper (U) and lower (L) halves of individual size-selected styles from plants of the *S_11_S_12_* line 314 left unpollinated, after incompatible (self) and compatible (×*S_13_S_14_*) pollinations were measured separately (C). Levels of S_11_-RNase were quantified by PhosphorImager and reported relative to total stylar RNase levels of unpollinated styles (D). Total stylar RNase levels (Σ) were calculated as the sum of upper and lower half measurements. Values are means±s.d. of S_11_-RNase levels from 2 independent experiments. Asterisks are significantly different (p<0.05, T test, n = 6).

Previous studies have shown that S-RNases enter both compatible and incompatible pollen tubes [16], [17]. Since the S-RNases are found only in the actively growing apical tip of the pollen tube [17], and because the styles harvested for analysis do not include the ovarian region, it was important to demonstrate that the pollen tips had not simply grown out of the stylar basis into the ovarian region thus removing S-RNase from the region analyzed. To measure the length of time needed for pollen tubes to reach the stylar base, flowers were pollinated with GFP-expressing pollen in a compatible cross. After five hours, the styles were removed as for S-RNase measurements, and placed in a humid atmosphere on a culture medium and the pollen allowed to continue growing. At 27 hours post-pollination, some fluorescence can be observed at the bottom of the styles, but the pollen tubes do not emerge until 28 hours post-pollination (Figure 3A). Entry of small numbers of pollen tubes (∼1% of the total number) into the stylar base *in vivo* 28 hours post-pollination was confirmed by aniline blue staining (Figure 3B). Since styles 24 hpp still contain the pollen tube tips, if S-RNase were translocated from the upper to the lower region of the style then S-RNase measurements should show a decrease in the upper region and a corresponding increase in the lower region of the style. To test this, styles were cut in half and the levels of the more abundant S_11_-RNase [41] were measured in upper and lower halves separately (Figure 3C). As seen with the S_12_-RNase, S_11_-RNase levels decrease 28% in fully compatible crosses (*S_11_S_12_*×*S_13_S_14_*) compared to the 15% decrease seen for incompatible (*S_11_S_12_*×*S_11_S_12_*) crosses (Figure 3D, white columns). Furthermore, S-RNase levels decrease in both upper and lower regions during fully compatible crosses, consistent with degradation rather than transport by the pollen tube tips (Figure 3D). The amounts of S_11_-RNase in the upper regions decrease to a greater extent than do at the lower regions, as expected given that the pollen tubes have not yet passed completely through the basal region of the style.

### Decreased stylar S-RNase levels correlate with stability of pollen mRNA and decreased stylar S-RNase mRNA

**Figure 4:**
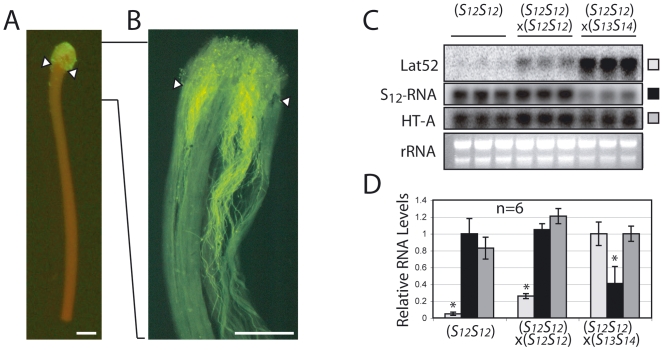
Pollen mRNA levels remain high and stylar S-RNase transcript levels decrease during compatible crosses. The stigma of *S_12_S_12_* line 2548 plants, observed twenty-four hours post-pollination with GFP-expressing pollen from transgenic *S_12_S_12_* plants, shows considerable amounts of GFP when observed by fluorescence microscopy (A), although actively growing pollen tube tips, as visualized by fluorescence microscopy of the fully incompatible cross (line 2548 selfed) stained with aniline blue, have entered the style (B). The stigmatic region (arrowheads) was removed before RNA extraction in order to measure pollen mRNA only in growing pollen tubes. All scale bars are 1 mm. Transcript levels using the indicated probes (pollen Lat52; stylar S_12_-RNase and HT-A) were measured in pools of twenty styles taken from the *S_12_S_12_* line 2548 either without pollination, after a fully incompatible cross (selfed) or after a fully compatible cross (×*S_13_S_14_*). The same amount of RNA as determined by OD measurement was loaded in each lane (C). Levels of each mRNA were quantified by PhosphorImager and reported relative to values in unpollinated styles (for S_12_-RNase) or in compatible crosses (for Lat52 and HT-A). Values are means±s.d. of the mRNA levels from 2 independent experiments. Asterisks are significantly different (p<0.05, T test, n = 6) (D).

Given the proposed cytotoxic role for the S-RNases, the decreased stylar enzyme levels in compatible pollinations should protect pollen mRNA against degradation. To test this prediction, the levels of the pollen-specific Lat52 mRNA were compared after fully incompatible (*S_12_S_12_*×*S_12_S_12_*) and fully compatible (*S_12_S_12_*×*S_13_S_14_*) pollinations of homozygous *S_12_* styles. At 24 hpp, the stigmatic region of the style is covered with both germinated and ungerminated GFP-labeled pollen (Figure 4A). While both germinated and ungerminated pollen contain Lat52 mRNA, the latter cannot take up stylar S-RNases and will produce a background signal making changes in mRNA levels of germinated pollen difficult to measure. Therefore, in order to restrict our analyses only to growing pollen tubes that have taken up the stylar S-RNases, the stigmatic surface of the style was excised just prior to RNA isolation. Aniline blue staining confirms that by this time, even incompatible pollen tubes have penetrated well inside the upper part of the style (Figure 4B). Lat52 mRNA is clearly more abundant in fully compatible compared to incompatible pollinations (Figure 4C), as expected based on the reduction in stylar S-RNase levels. We note that at 24 hpp, Lat52 mRNA levels in incompatible crosses are 25% those in compatible crosses, a decrease similar to that found using radiolabeled pollen rRNA in *Nicotiana* [43]. S-RNases thus appear to degrade ribosomal and messenger RNA indiscriminately *in vivo*.

**Figure 5:**
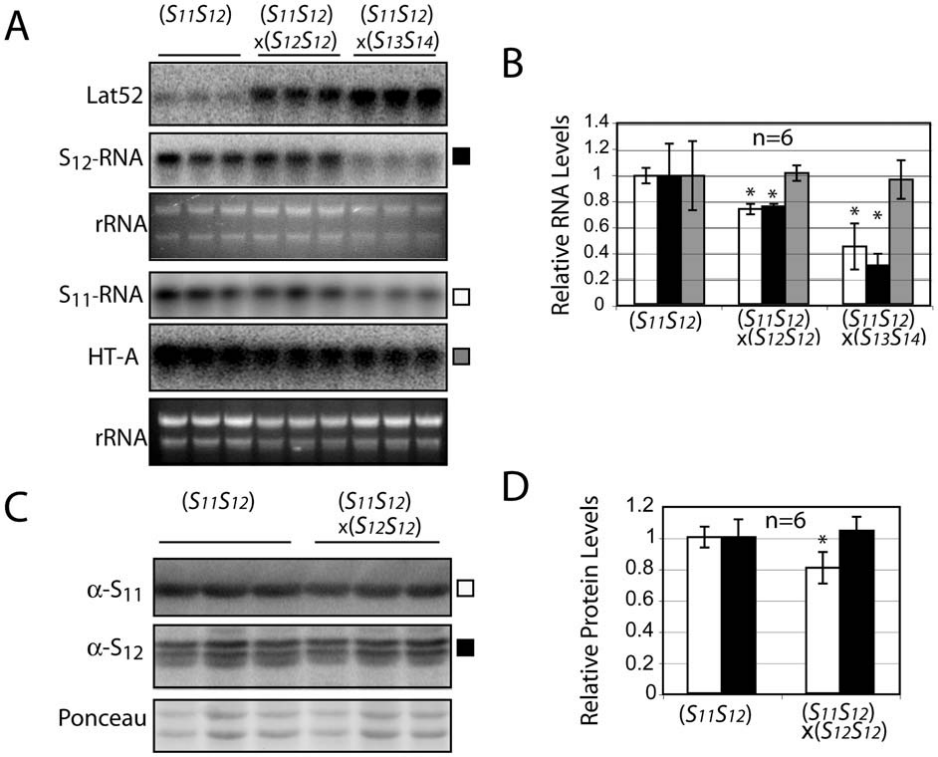
Reduced levels of non-self S-RNases are not due to a reduction in stylar S-RNase transcript levels. Levels of mRNA (pollen Lat52; stylar S_12_-RNase, S_11_-RNase and HT-A) were measured in pools of twenty styles taken from the *S_11_S_12_* line 314 either without pollination, after pollination with incompatible *S_12_* pollen (*S_11_S_12_*×*S_12_ S_12_*) or after a fully compatible pollination (*S_11_S_12_*×*S_13_ S_14_*) with *S_13_*, *S_14_* pollen. Similar amounts of RNA were loaded in each lane (A). Levels of mRNA were quantified by PhosphorImager and reported relative to values in unpollinated styles (for S_11_- and S_12_-RNases) (B). Values are means±s.d. of transcript levels from 2 independent experiments. Asterisks are significantly different (p<0.05, T test, n = 6). S-RNase levels were measured by western blot in size-selected styles of line 314 either left unpollinated or pollinated with S_12_ pollen (*S_11_S_12_*×*S_12_S_12_*) (C). PhosphorImager quantitated S-RNase protein levels are reported relative to values in unpollinated styles. Values are means±s.d. from 2 independent experiments. Asterisks are significantly different (p<0.05, T test, n = 6) (D).

Unexpectedly, however, compatible pollinations 24 hpp also resulted in a 60% decrease in S-RNase mRNA levels in the styles themselves. This phenomenon is restricted to the S-RNase RNA and is not observed with levels of the stylar HT-A mRNA, a gene outside the S-locus that serves here as a control for general RNA levels within the style (Figure 4C, D). We were immediately curious to know if the decrease in stylar S-RNase mRNA levels might explain the decrease in stylar S-RNase protein observed during compatible crosses, as this would directly contradict our conclusion that the S-RNase protein was being specifically degraded. To test this, additional crosses were performed using styles expressing both S_11_- and S_12_-RNases (*S_11_S_12_*). Molecular analyses of these styles recapitulate the essential features observed in homozygous *S_12_* styles, with pollen-specific Lat52 mRNA once again more abundant in fully compatible (*S_11_S_12_*×*S_13_S_14_*) compared to incompatible crosses (*S_11_S_12_*×*S_12_S_12_*) (Figure 5A, upper panel), HT-A mRNA remaining unchanged during both types of crosses (Figure 5B, lower panel), and stylar S-RNase transcript levels decreasing by 25% and 60–70% during incompatible (*S_11_S_12_*×*S_12_S_12_*) or compatible (*S_11_S_12_*×*S_13_S_14_*) crosses, respectively (Figure 5B, black and white bars). However, when S-RNase protein levels are examined, compatible S_11_-RNase levels decrease by 20% whereas the self S_12_-RNase levels remain constant (Figure 5C, D). To understand this experiment, it is first necessary to recall that during incompatible crosses (*S_11_S_12_*×*S_12_S_12_*), both a genetically compatible S_11_-RNase and a genetically incompatible (self) S_12_-RNase will enter the *S_12_* pollen tubes. These two different S-RNases respond differently in the *S_12_* pollen tubes, leading to the conclusion that the individual pollen tubes can distinguish between them. However, decreased transcript levels are observed for both S_11_- and S_12_-RNases mRNAs in the styles, indicating that the specific signal apparently associated with a compatible reaction in the pollen can be read by styles even in the presence of an on-going incompatible reaction. More importantly, we conclude from the observation that S_11_-RNase mRNA levels decrease but S_11_-RNase levels do not that the reduction in levels of non-self S-RNases are not simply due to a reduction in their transcript levels in the style.

**Figure 6:**
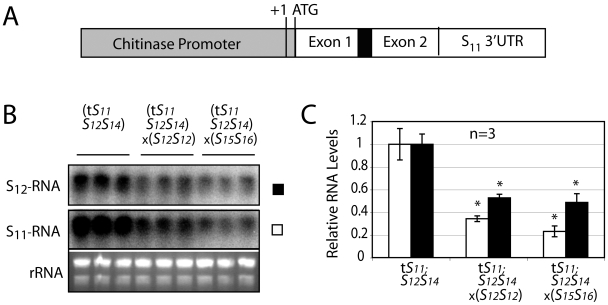
Compatible pollen triggers a decrease in S-RNase transcript levels by a post-transcriptional mechanism. (A) An S_11_-RNAse transgene containing the entire S11-RNase coding sequence and 3'UTR (white) under control of the style-specific chitinase gene promoter and 5'UTR (gray). (B) Northern blot analysis of *S_12_S_14_* plants expressing the transgenic S_11_-RNase, either without pollination, after a fully incompatible cross (×*S_12_S_12_*) or after a fully compatible cross (×*S_15_S_16_*) using gene probes against either the S_11_- or the S_12_-RNase transcript. (C) Levels of both S_11_- and S_12_-RNase transcripts (white and black bars, respectively) were quantified by PhosphorImager and reported relative to unpollinated styles. Values are means±s.d. of S-RNase transcript mRNA levels. Asterisks are significantly different (p<0.05, T test, n = 3).

A reduction in stylar S-RNase transcript levels as observed here could result from either a decrease in the rate of synthesis, an increase in the rate of degradation, or both. To evaluate the effect of RNA production, we compared levels of S_11_-RNase transcript produced from a heterologous chitinase promoter (Figure 6A) with the levels of an authentic S_12_-RNase transcript. We observe a decrease in levels of both transcripts (Figure 6B), clearly indicating active degradation of S-RNase transcripts. It is intriguing that despite the initially higher levels of S_11_-RNase transcript produced using the heterologous promoter, similar amounts of both S_11_- and S_12_-RNase are found after fully compatible crosses. The apparent rate constant for degradation thus appears greater for the S_11_-RNase transcript. Unfortunately, experiments designed to address the mechanism of degradation by testing for induction of small RNAs complementary to the 3′ UTR of the S-RNase transcript were unsuccessful.

## Discussion

In this study, we have examined the molecular events occurring in both pollen and style during compatible and incompatible crosses. We find that during incompatible crosses, where pollen tube growth is blocked mid-style and in consequence is unable to fertilize the ovules, S-RNase protein levels are virtually identical to those found in unpollinated styles (Figure 2D) and the amount of pollen RNA is less abundant compared to fully compatible crosses (Figure 4C,D). This reinforces the view that pollen rejection and RNA degradation in the pollen are causally linked by S-RNase activity. In contrast, compatible crosses show markedly decreased levels of stylar S-RNase (Figure 2D) and higher levels of pollen RNA (Figure 4C,D). We have shown that S-RNase levels in the styles do not decrease because growing pollen tubes sequester and move the S-RNase into the stylar base and out of the style (Figure 3). Instead, S-RNase levels appear to decrease by an active degradation process. Unfortunately, we did not observe intermediate forms of the degradation, such as higher molecular weight ubiquitinated forms. There are two factors which likely contribute to this. First, the bulk of the stylar S-RNase remains extracellular, making it difficult to detect small amounts of the ubiquitinated form. Second, the half-life of the ubiquitinated intermediates may be quite short, further reducing steady state values of the degradation intermediates.

The results shown here also allow S-RNase degradation following compatible pollinations to be distinguished from a simple failure to accumulate S-RNase protein. This distinction is important since S-RNase levels do normally increase as a function of the age of the style (Figure 1), and is most easily observed from crosses involving a diploid *S_11_S_12_* style with incompatible *S_12_* pollen from a homozygous *S_12_S_12_* plant (Figure 5D). In these crosses, only the non-self S_11_-RNase is degraded whereas levels of the self S_12_-RNase remain constant. Since the levels of S-RNase transcript for both alleles decreases, any failure to accumulate S-RNAse protein would be expected for both. This is clearly counter to our experimental observations showing haplotype specific changes in S-RNase protein levels.

One possible explanation for the decrease in stylar S-RNase transcript abundance following pollination is that these changes may be due to flower senescence. Indeed, senescence was proposed to explain the concurrent decrease in levels of both S-RNase and HT-A transcripts in 2-days old S. chacoense styles, either unpollinated or after compatible pollinations [31], [32], with a delay in floral senescence following incompatible pollinations explaining the maintenance of high S-RNase and HT-A transcript levels. However, our results with 1-day old styles differ slightly in that S-RNase and HT-A transcript levels respond differentially to compatible pollination, suggesting that at shorter times post-pollination, effects of senescence and pollination may be distinguished. Interestingly, pollination-induced changes in the flower include changes in transmitting tract cell morphology [44] that are visible within a day post-pollination, while responses to the senescence hormone ethylene involves changes in the entire flower. Furthermore, while both pollination and ethylene have effects on transcripts within transmitting tract cells [45], only pollination and not ethylene was able to induce differential effects on stylar transcript abundance. The transcript-specific nature of changes in RNA levels were taken to indicate a result of pollination rather than senescence, and we draw the same conclusion from the difference in S-RNase and HT-A transcript levels (Figure 4). Interestingly, ethylene production following pollination occurs in two bouts, one several hours post-pollination that is independent of the type of pollination and a second about one day post-pollination that occurs only in compatible pollinations [46]. This second bout of ethylene production is thus a good candidate for induction of senescence, and could possibly explain the morphology of flowers following compatible crosses two days post-pollination (Figure S1). Taken together, we conclude our results can not be adequately explained by flower senescence, although further studies on the effects of ethylene and the timing of senescence in our system will be required to fully document this view.

**Figure 7:**
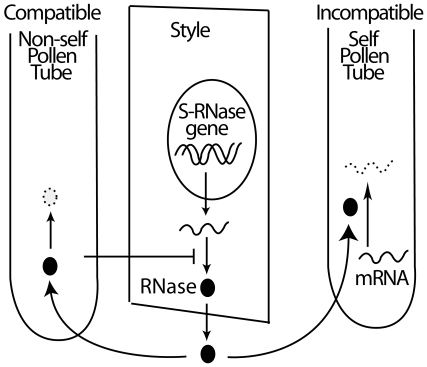
Model illustrating reciprocal pollen-style interactions during compatible crosses. Degradation of a non-self S-RNase inside compatible pollen tubes helps protect pollen tube RNA from degradation. Reciprocal signaling to stylar cells decreases S-RNase mRNA, thus reducing the ability of the style to renew the extracellular S-RNase pool. In contrast, high levels of S-RNase in self pollen tubes lead to pollen mRNA degradation.

These results reveal the existence of a previously unsuspected feedback loop in the complex communication between pollen and styles in compatible crosses. Signaling from pollen to style has been inferred from changes in flower morphology that become apparent by two days post pollination, where the petals close up and prepare to drop off allowing the fruit to develop unencumbered (Figure S1). However, our results identify changes in stylar gene expression that clearly precede these morphological changes during compatible pollinations. We propose that the signal for lowering S-RNase transcript levels in the style is generated by pollen tubes containing non-self (compatible) S-RNases and suggest that this decreased transcripts level results in a decreased ability to replenish the S-RNase pool, thus reinforcing the compatible reaction (Figure 7).

Previous RNA expression analyses performed 48 h following a compatible pollination on styles of *S. chacoense* have revealed a decrease of S-RNase transcripts [32], similar to what we have observed in this study. Curiously, these authors also noted a decrease in HT-A transcripts levels in styles 48 h after compatible pollinations. Since our observations were only made 24 h post pollinations, it is possible that a decrease in HT-A transcripts might take place between 24 h and 48 h, which would then correspond to the observations of O'Brien and colleagues. More recently, transcripts profiles of the S-RNase gene in the SI cultivar “Xinshiji” of apricot, *Prunus armenica*, have been reported, using a high-flux real-time FQRT-PCR method [47]. The expression profile of the S-RNase gene 24 h after compatible pollinations decreased, similar to what we observed with *S. chacoense*. Surprisingly, however, these authors also observed a sharp decrease in S-RNase gene expression in unpollinated styles, and only in cross-pollinated styles did the S-RNase transcripts remain high. While these results in *Prunus* show that S-RNase transcript levels behave similarly to *S.chacoense* in self and non-self crosses, the mechanism is presumably different with transcription of S-locus genes somehow activated by non-self pollen [47].

Interestingly, our results may provide an explanation for the “mentor pollen” effect, which has been exploited with some success to overcome self- and inter-specific incompatibility (for references see [1], [2]). The mentor pollen effect occurs after pollinations with either inactivated-compatible (mentor) pollen or a mixture of mentor and incompatible pollen, followed one or two days later by incompatible pollinations, and results in fruits set from the incompatible pollen [48], [49]. According to our model, the mentor pollen would prepare the style for a subsequent incompatible pollination by not only decreasing the stylar S-RNase load, but also by impairing the ability of the style to synthesize more S-RNase. Cases where a mentor pollen effect are not observed [1], [2]) may be due to very high S-RNase levels that still exceed threshold [40], [41] even after passage of mentor pollen.

Our model may also be thought of in terms of resource allocation [50], as the stylar response to compatible pollen could result in a shift from synthesis of SI system components to synthesis of other components required for success of compatible pollinations. In contrast, incompatible pollen not only contains an S-RNase active in pollen RNA degradation, but is also unable to modulate gene expression in the style.

The idea that feedback mechanisms contribute to regulation of the SI response is not without precedent. In a recent model for SI in *Nicotiana*, it has been suggested that pollen rejection involves a complex interplay between the levels of the stylar protein HT-B, proposed to be required for S-RNase release into the cytoplasm, and the maintenance in the pollen cytoplasm of a protein required for HT-B degradation [29]. During incompatible crosses, release of S-RNase into the pollen cytoplasm will result in decreased pollen mRNA, similar to what we observe with *S. chacoense*, and thus a decreased ability of the pollen to replenish HT-B degrading factors. As a result, this positive feedback mechanism ensures that levels of S-RNase in the pollen tube cytoplasm, and the ensuing pollen rejection, will proceed to completion. An analogous positive feedback is observed here during the compatible crosses, as not only is S-RNase degraded in the pollen tubes, but also the ability of the style to manufacture more S-RNase should be compromised by the decreasing S-RNase transcript levels. This positive feedback thus facilitates the growth of compatible pollen tubes through the style. Interestingly, mathematical modeling of positive feedback mechanisms in the cell cycle suggests that feedback can function to produce a switch-like behavior, where, for example, entry into mitosis is either all-or-nothing [51], and the metaphase to anaphase transition is abrupt rather than gradual [52]. This switch-like, all-or-nothing behavior is also characteristic of pollen rejection, and it is revealing that pollen rejection and acceptance may thus both involve feedback mechanisms.

It is intriguing that while entry of S-RNases into pollen tubes has been shown microscopically for both *Nicotiana* [16] and *S. chacoense* [17], degradation of S-RNases has not been detected in compatible crosses of *Nicotiana*. Clearly, there are mechanisms in addition to degradation that can be used to restrict RNase activity. This is likely to occur in *S. chacoense* as well, as we have previously observed that S-RNases enter both compatible and incompatible pollen tubes [17]. This suggests that a multistep mechanism involving either sequestration or RNase inhibition in addition to S-RNase degradation is part of the SI response in this species. The SI reaction is now appearing more complex than previously thought.

## Methods

### Plant Materials

The diploid (2n = 2x = 24) self-incompatible genotypes of *Solanum chacoense* used in this study include the genetic lines PI458314 (*S_11_S_12_*) and PI230582 (*S_13_S_14_*) described previously [53], as well as line PI458312, fully compatible with the two previous lines (*S_15_S_16_*). These parental lines, obtained from the Potato Introduction Station (Sturgeon Bay, WI), were used in the production of line G4 (*S_12_S_14_*) [54] and line 2548 (*S_12_S_12_*) [41]. Transgenic lines include line T31 (t*S_11_-S_12_S_14_*; a transgenic G4 expressing the *S_11_-*RNase gene from the style specific Chitinase promoter) [9], and a range of different S-genotypes expressing GFP under the control of the pollen-specific Lat52 promoter, (kindly provided by Dr. S. McCormick, UC Berkley) including 2548 (tGFP-*S_12_S_12_*), G4 (tGFP-*S_13_S_14_*) and line 1022 (tGFP-*S_11_S_13_*) (latter kindly provided by Dr. Matton, Université de Montréal).

### Genetic Crosses and observation of pollen tube growth in styles

Crosses were scored as fully compatible when almost all pollinations set fruit, or as fully incompatible if pollinations never formed fruits. To ensure uniformity in the stage of flower development, all open flowers were removed in the afternoon preceding crosses. Flowers open during the early morning and are thus day 0 flowers. All excised styles were measured, using a slide gauge, at the collecting time and only those with the same length and diameter were retained for the analyses. Pollen for genetic crosses was freshly collected from plant material grown in greenhouses under natural lighting.

Pollen tube growth in the styles was monitored using aniline blue staining [9]. For *in vitro* culture experiments, the pollinated styles were excised 5 hours post pollination and inoculated on slides with a solid medium containing 100 mg/L H_3_BO_4_, 300 mg/L CaCl_2_, 10% sucrose and 1% agarose. The slides were then placed in a container and kept in a growth room at 22±2°C under dark condition. To observe pollen tube germination and growth *in vivo*, styles crossed with pollen expressing GFP were observed by fluorescence microscopy (ZEISS, SteREO Discovery V12).

### Protein analysis and immunological quantification of S-RNase levels

The extraction and analysis of protein from individual styles was carried out as described [40], [41] using rabbit polyclonal antibodies specifically directed against the S_11_-RNase HVa region (peptide KPKLTYNYFSDKMLN) [8] or the S_12_-RNase HVb region (peptide TTEVESKKNQFFWXK) [55]. Rabbit anti-GFP (GeneTex Inc. Texas, 78245 USA) and ^125^I labeled goat anti-rabbit (0.5 µCi/mmol; Perkin-Elmer) were obtained commercially. All immunological quantification used proteins transferred electrophoretically to Hybond-P membranes (Amersham Biosciences), incubated with the radiolabeled secondary antibody, exposed for 1–3 days at room temperature with a Phosphor screen, and the screen imaged using a PhosphorImager scanner (Typhoon 9200, Amersham Bioscience). Data on the scanned images were quantified using the software supplied by the manufacturer and analyzed by statistical methods.

### Northern blots and RNA quantification

Pools of 20 pollinated or unpollinated control styles were collected 24 hours after the pollination, frozen in liquid nitrogen and stored in −80°C until use. Total stylar RNA was extracted and purified using the RNeasy plant mini kit (Qiagen) and the quantity and quality assessed by the ratio of OD at 260/280 nm. Northern blot analyses using Hybond-N+ membranes (Amersham Pharmacia Biotech) were performed as described [56] using 5 µg total RNA for each lane. Probes for Northern blots were prepared from DNA fragments amplified by RT-PCR using Lat52 F1279 (5′-GTGCAACTGTGAAGTTGC-3′) and Lat52 R1610 (5′-TTA GAA TTC AAG GGG GAA TA-3′) to generate a 332 bp Lat52 probe from tomato [57]; S11 F1230 (5′- CTATTCAGTGTAAGCA-3′) and S11 R1554 (5′-GGACGAAAAAATATTTTC-3′) to generate a 325 bp S_11_-RNase probe from *S. chacoense* [58]; S12 F391 (5′- TAACTTGACCACCACCG-3′) and S12 R766 (5′-GTCATGGAAATGTAACCC-3′) to generate a 376 bp S_12_-RNase probe from *S.chacoense* [55]. A 500 bp HT-A probe was synthesized using the entire coding region of a ScHT-A1 cDNA from *S. chacoense* [32]. All probes were prepared using the Strip-EZ PCR Kit (Ambion, Texas) to facilitate reuse of the blots for multiple probes. All radioactive membranes were imaged and quantitated using a PhosphorImager scanner (Typhoon 9200, Amersham Bioscience).

## Supporting Information

Figure S1 Morphological changes are observed in flowers two days after compatible pollinations. Flowers were photographed daily after crosses with compatible pollen (Com), incompatible pollen (Inc) or when left unpollinated (Unp) as controls. Morphological changes seen two days (2 d) after compatible crosses do not occur when flowers are not pollinated or are crossed with incompatible pollen.
(9.09 MB TIF)
