# Supplementary figures and images for: Compatible Pollinations in Solanum chacoense Decrease Both S-RNase and S-RNase mRNA
Source: PLoS One. 2009 Jun 3;4(6):e5774. doi: 10.1371/journal.pone.0005774 (PMC2686617; doi:10.1371/journal.pone.0005774)

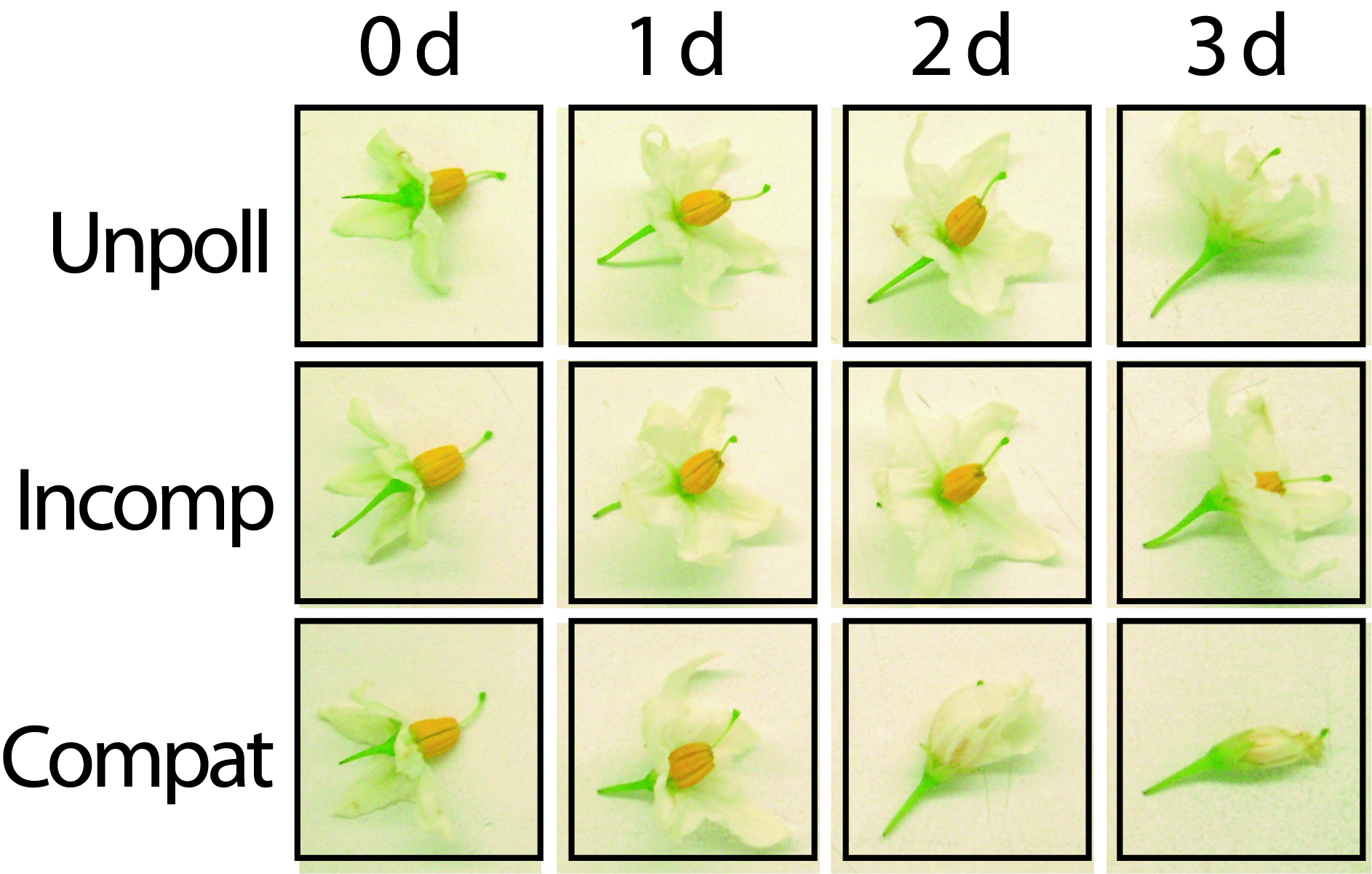

Supplement: Figure S1 — Morphological changes are observed in flowers two days after compatible pollinations. Flowers were photographed daily after crosses with compatible pollen (Com), incompatible pollen (Inc) or when left unpollinated (Unp) as controls. Morphological changes seen two days (2 d) after compatible crosses do not occur when flowers are not pollinated or are crossed with incompatible pollen. (9.09 MB TIF) [file pone.0005774.s001.tif]
